# Vision-Based Apple Classification for Smart Manufacturing

**DOI:** 10.3390/s18124353

**Published:** 2018-12-10

**Authors:** Ahsiah Ismail, Mohd Yamani Idna Idris, Mohamad Nizam Ayub, Lip Yee Por

**Affiliations:** Department of Computer System and Technology, Faculty of Computer Science and Information Technology, University of Malaya, 50603 Kuala Lumpur, Malaysia; ahsiahismail15@siswa.um.edu.my (A.I.); nizam_ayub@um.edu.my (M.N.A.); porlip@um.edu.my (L.Y.P.)

**Keywords:** vision sensor, smart manufacturing, image recognition, Bag of Words, Spatial Pyramid Matching, Convolutional Neural Network

## Abstract

Smart manufacturing enables an efficient manufacturing process by optimizing production and product transaction. The optimization is performed through data analytics that requires reliable and informative data as input. Therefore, in this paper, an accurate data capture approach based on a vision sensor is proposed. Three image recognition methods are studied to determine the best vision-based classification technique, namely Bag of Words (BOW), Spatial Pyramid Matching (SPM) and Convolutional Neural Network (CNN). The vision-based classifiers categorize the apple as defective and non-defective that can be used for automatic inspection, sorting and further analytics. A total of 550 apple images are collected to test the classifiers. The images consist of 275 non-defective and 275 defective apples. The defective category includes various types of defect and severity. The vision-based classifiers are trained and evaluated according to the *K*-fold cross-validation. The performances of the classifiers from 2-fold, 3-fold, 4-fold, 5-fold and 10-fold are compared. From the evaluation, SPM with SVM classifier attained 98.15% classification accuracy for 10-fold and outperformed the others. In terms of computational time, CNN with SVM classifier is the fastest. However, minimal time difference is observed between the computational time of CNN and SPM, which were separated by only 0.05 s.

## 1. Introduction

Smart manufacturing employs a high level adaptability of computer control and various technologies into the existing manufacturing process in optimizing the productivity. Data analytics in smart manufacturing can shift the manufacturing process from a reactionary to a predictive practice. The predictive analytics is important for future planning and to improve the future production. Data analytics are capable of identifying the patterns and learning from the current production data for future decision-making and actions. The data can be acquired via sensors at any level of the manufacturing process. The acquired data are transmitted, shared and exchanged to improve the product quality and the production productivity.

For this reason, a vision-based apple classification for smart manufacturing is proposed. The vision-based apple classification is proposed to differentiate between defective and non-defective apples for automatic inspection and sorting processes. Further data analytics can also be performed based on the current production data of the defective and non-defective fruits in apple production. The analytics process identifies the patterns and learns for future planning and prediction; which helps improving the apple growth and processing efficiency. In the proposed system, visual sensors are used for data acquisition. This paper will focus on the image processing for vision-based apple classification. The vision-based apple classifications are designed based on dictionary-based features (Bag of Words (BOW) and Spatial Pyramid Matching (SPM)) and CNN-based features (Convolutional Neural Network (CNN)). The BOW, SPM and CNN methods are evaluated to determine the best vision-based classification for detecting defective and non-defective apples in the proposed system, as it contains more discriminative and structural information compared to being solely dependent on the local features representation such as keypoint-based features. To test the proposed system, a dataset containing a variety of apple images taken from various angles are created. The images of stem end and calyx are also included. The apples in the dataset consist of non-defective and defective apples with different types of defect, severity, region and location of the defects. The performance of each method in the proposed vision-based apple classification are compared in terms of sensitivity, specificity, accuracy and computational time using *K*-fold cross-validation.

This paper is organized as follows: Section 2 presents the related works on the image recognition methods which are used to differentiate between defective and non-defective apples. Details of the proposed work are described in Section 3, that includes an overview of the proposed vision-based apple classification. Section 4 reports the results. The performance of the proposed method is discussed in Section 5. Finally, the conclusions and future works are highlighted in Section 6. 

## 2. Related Works

In computer vision, attaining the capability of human recognition remains a challenge [1,2,3]. However, continuous development in the field has led to the advancement of image recognition technology. Image recognition is becoming more reliable with consistent performance. This opens the opportunity for a real-world implementation such as implementing image recognition in the inspection processes of the manufacturing industry to detect and classify defective and non-defective products. Image recognition is related to image processing to detect the instances of the object in digital images. To detect the object using an image recognition method, it is become a necessary to define the features, then use classifier techniques to classify the object. The features are the measurable characteristic of the object and the classifier categorize the features into classes according to their similarity. Generally, image recognition methods classify the object based on features such as shape, color, texture, etc. This paper will focus on other feature representations which are keypoint-based features, dictionary-based features and CNN-based features. 

The keypoint-based features describe the image by locating the patches around the keypoint descriptor which is centered on the keypoint. Harris corner detection [4], Scale Invariant Feature Transform (SIFT) [5], Speeded up Robust Features (SURF) [6,7] and Accelerated Segment Test (FAST) [8] are among the keypoint-based features considered in image recognition. Harris detection has been shown to be robust in matching with good stability properties and repeatability [9]. However, the Harris detector is sensitive to scale changes. Contrarily, the SIFT detector and descriptor [5] are invariant to the changes of scale and rotation. Also, the SIFT detector and descriptor are robust to affine distortion and illumination changes [5]. The SIFT detector has demonstrated a high repeatability and accuracy, but the high dimensionality of the SIFT descriptor leads to a high computational cost [10]. The issue of the high computational cost in SIFT is addressed in SURF. SURF is faster compared to SIFT without degrading the quality of the detected points and more robust to noise than the SIFT method [11]. However, both SIFT and SURF are unsuitable for real-time applications due to their high computational complexity [12]. Rosten et al. [8] introduce FAST to improve the computational time of earlier methods. Loncomilla et al. [13] reported that FAST is faster compared to SIFT and SURF, however, the FAST method is not invariant to scale [13]. The keypoint-based features are extensively used in image recognition due to their various advantages. However, the limitations of the keypoint-based features are that different contexts or scenes can be represented by a similar descriptor and a patch of context or scene can be represented by different descriptors due to noise or distortion [14].

To overcome this limitation, researchers widely use dictionary-based features for image recognition. Dictionary-based features describe the images by counting the frequency of occurrence for each visual pattern (visual words) and used it as a feature for training the classifier. The BOW is a well-known dictionary-based feature method. It is easy to implement [15,16], robust to object occlusion, image clutter, non-rigid deformation and also robust to viewpoint changes [17]. However, BOW disregards the spatial information in the visual words, which is an important element in object recognition and classification tasks [18]. This leads to missing information on the image composition and the features spatial arrangement [18]. The aforementioned issues of the BOW method are then addressed in the SPM method proposed by Lazebnik et al. [19]. SPM is the most established extension of the orderless BOW method and a popular approach for encoding local and global spatial information in visual words [20]. The SPM method adds spatial information in the unstructured BOW model to improve the image representation and better distinguish objects. This spatial information is important to discriminate between objects because different objects may have the same visual appearance with different spatial distributions [21]. However, the SPM method generates large numbers and massive redundancies of feature vectors with high dimensionality [20]. Thus, it requires high computing resources. To effectively generate more compact feature vectors, Penatti et al. [20] proposed Word Spatial Arrangement (WSA) which skips the pre- and post-processing stage for spatial verification. On the other hand, to eliminate the image feature redundancy, Lin et al. [18] introduced a framework, called iterative keypoint selection (IKS) to select the representative keypoints. Similarly, Li et al. proposed SPM-PCA [22] to resolve this limitation. Xie et al. also proposed a new spatial partitioning scheme to avoid feature redundancy based on a modified pyramid matching kernel [23] which also accelerates the computational time.

In recent literature starting from 2015, deep learning technology image recognition methods such as the CNN method have been rapidly developed. The CNN method had been successfully tested on various datasets such as NORB [24], MNIST [25] and ImageNet [26]. The major issue of the CNN method is that it requires a fixed-size input image [27]. To address this issue, He et al. proposed a new network structure (SPP-net) which can generate a fixed-length representation regardless of image size or scale [27]. Another disadvantage of the CNN method is that as the network depth in the CNN structure increases, the neural network becomes more challenging to train and the gradients are often prone to vanish or explode [28,29]. To ease the training of deeper networks, He et al. [28] proposed a residual learning framework to explicitly reformulate the layers as learning residual functions. Another disadvantage of the CNN method is that it is computationally expensive and requires a large number of images for training to avoid over-fitting [27,29,30].

In this paper, the dictionary-based features (BOW and SPM) and CNN-based features are used in the proposed vision-based apple classification to determine the best image recognition method for defective and non-defective apple classification in smart manufacturing. Of all the methods discussed earlier, the dictionary-based features and CNN-based features approaches produce more “semantics-oriented” result in recognizing objects as it contains much more discriminative and structural information compared to being solely dependent on the local features representation such as keypoint-based features. 

## 3. Materials and Methods

**Figure 1:**
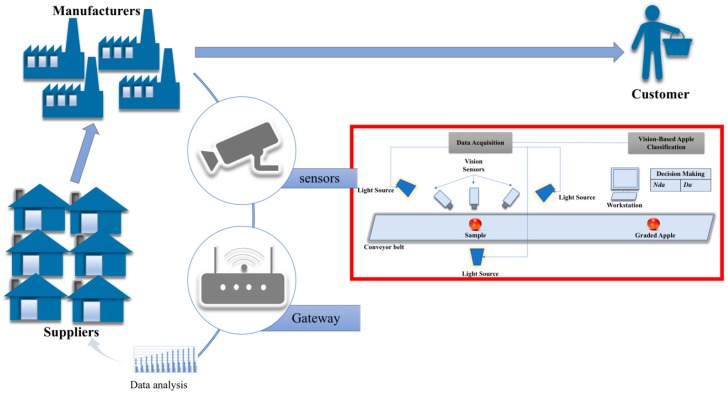
Illustration of the proposed vision-based apple classification for smart manufacturing which employs visual sensors for visual data acquisition (defective apple (*Da*) and non-defective apple (*Nda*)).

The smart manufacturing system for the proposed vision-based apple classification is illustrated in Figure 1. The vision-based apple classification consists of two main sections, namely data acquisition and vision-based apple classification. The data acquisition captures the images of the apples placed on the conveyor belt via visual sensors. To ensure the quality of the images, light sources are placed near the vision sensors. The images of the apples are then sent to the vision-based apple classification section. This section is where the proposed work is applied, wherein the images of the apples are used as input to the image classification method followed by their classification. The classification output from the classifier will sort the apples into the appropriate bins according to their category which is defective or non-defective. Based on the current production data of the defective and non-defective fruits in apple production, the data analytics process will identify the patterns and learn for future decision making and actions.

**Figure 2:**
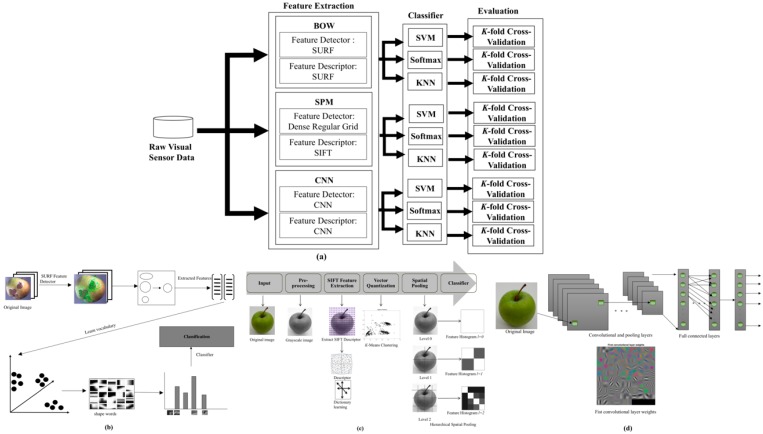
Vision-based apple classification (**a**) Overall scheme with three different image recognition methods (**b**) BOW, (**c**) SPM and (**d**) CNN.

Generally, the proposed work can be divided into two phases, training and testing. In this paper, three feature extraction methods are evaluated which are the BOW, SPM and CNN methods, which have state-of-the-art classification performance to classify the apples between the non-defective and defective. Then, the extracted features from these methods are classified with three classifiers, namely SVM, Softmax and K-Nearest Neighbors (KNN) to obtain the most suitable classifier for all the feature extraction methods. The overall scheme for the proposed vision-based apple classification system is shown in Figure 2a. Each of these methods will be discussed in the following subsections.

### 3.1. Feature Extraction

#### 3.1.1. BOW Method

The structure of the proposed vision-based apple classification system using the BOW method are shown in Figure 2b. In the BOW method, the SURF feature extraction technique is used since it is the fastest feature extraction method. In BOW, the apple images are described by visual patterns (visual words). The words obtained in BOW method are more general than low-level descriptors since the descriptors are quantized into “similar looking” regions in the clustering step in BOW.

#### 3.1.2. SPM Method

The SPM method implemented in the proposed vision-based apple classification system is shown in Figure 2c. As can be seen in Figure 2c, the SPM method consists of five stages namely pre-processing, feature extraction, vector quantization, spatial pooling and classifier. In SPM method, the pre-processing stage converted all the apple images into grayscale images to reduce the dimension and simplified the data.

In the feature extraction stage, the SIFT feature descriptor is used to extract feature vectors from all over the image on a regular grid. Each grid representing a patch of 16 × 16 pixels and overlap by 8 pixels with its neighbours. The patch is further divided into 4 × 4 cells wherein the gradient orientation of 8-bins histogram is computed for each cell. The 8-bins histogram from all cells are then combined, resulting in a total of 128-element vector representing a patch. The descriptors obtained are invariant to scale, orientation, rotation and partially invariant to affine distortion and illumination change.

**Figure 3:**
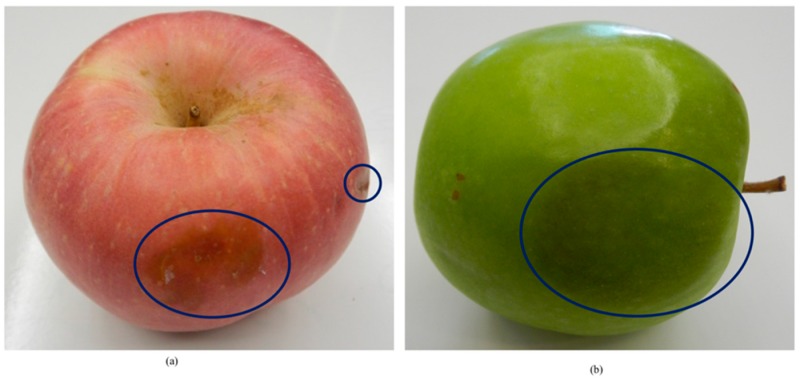
Examples of bruising defects (**a**) red skinned apple and (**b**) green skinned apple.

The normalization step in the SIFT descriptor is skipped to deal with the low-contrast region when the patch has a weak gradient magnitude. This is to avoid misclassification of unobvious defective apples such as ones with discoloration defects on the apple skin. Examples of the low contrast region on the apple skin that caused by a bruising defect are shown in Figure 3. The defect appears as a darker pigmented area compared to the healthy region.

Then, the extracted feature vectors are clustered into a 500 visual word vocabulary to create a codebook using the K-Means clustering algorithm in the vector quantization stage. The codebook is formed using the K-Means clustering with a total of 100 iterations. In this algorithm, each feature will be clustered according to the distance value. The features are inserted into a cluster that has a minimum distance from the center, thus grouping it into a particular cluster. 

The spatial pooling stage transforms the coded features into the global image representation [31,32,33]. In this work, the pyramid matching is built using spatial pooling in two-dimensional image space with vector quantization technique. Spatial pyramid is the multi-level recursive image decomposition method. The image is divided into a sequence of grids according to pyramid level. Then the features extracted from the entire cell grid are concatenated forming the feature vector. Thus, the feature vector obtained is the collection of orderless feature histograms computed over grid cells. Specifically, all the feature vectors are quantized into *M* discrete types. Each of the *m* channels gives two sets of two-dimensional vectors which are *Xm* and *Ym.* The *Xm* and *Ym* represent the features of type *m* coordinates found in the respective images. Then, the final kernel sums the separate channel kernels:

(1) $$K_{L}\left(X,Y\right)=\;\sum_{m=1}^{M}K_{L}\left(X_{m},Y_{m}\right),$$

This approach gives the advantage in maintaining continuity with the “visual vocabulary” paradigm. In this experiment, the regular spatial pooling in SPM method with two pyramid levels is used to partition the pyramid level. The two pyramid levels (L=2) that were used in this work are according to the experimental setup used in a previous study [19,34,35,36]. The previous settings used L=2 with vocabulary size of M=200 and were tested on a small resolution image of about 300 × 250 pixels [19]. However, in this work two pyramid levels with the settings of L=2, M=500 and the patch size of 1500 are used. This is to deal with the high resolution images in the dataset. As depicted in Figure 2c, the decomposition of an image consists of a single cell at level 0. The image representation in level 0 is equal to the standard BOW method. At the top level, which is level 0, the SPM extracts the features from the apple image. Then, the image is divided into four quadrants at level 1 and nine grid cells at level 2. Each spatial cell consists of a feature vector and will produce one feature histogram.

#### 3.1.3. CNN Method

The basic structure of the CNN method used in the proposed work is shown in Figure 2d. The CNN structure consists of input layer, convolutional and pooling layers, full connected layers, output layer and classification. During the process convolution for a certain layer, the filter slides over that layer and the weight matrix of that layer does Hadamard product with the pixel values below the filter.

### 3.2. Classifier

The features extracted from the three methods are classified using three classifiers namely; SVM, Sofmax and KNN. Their performance are evaluated and compared.

#### 3.2.1. SVM Classifier

Support Vector Machine (SVM) [37] is employed in all prior mentioned feature extraction methods to classify the apple images into defective and non-defective. The SVM technique is chosen due to its high accuracy performance and well established efficiency in many image recognition fields [38,39,40,41,42]. To optimize the performance of SVM, the libsvm [43] grid search algorithm is used to determine the kernel parameters. For multi-class classification in SVM, a one-against-one rule is used. In the one-against-one rule, the classifier learns to classify two classes (defective and non-defective apples). Each one of the classes was trained to separate between these two classes. Then, a test sample of the apple images was input to the classifier and the test sample is assigned to the class according to the highest respond label obtained from the classifier. The procedure of SVM classification is described as follows:

*Step 1*: Convert the apple dataset to the SVM package format: The SVM package requires each of the data instances are represented in the form of vectors of the real numbers.

*Step 2*: Apply the scaling on the data $K\left(x,y\right)=e^{-\gamma {\left|\left|x-y\right|\right|}^{2}}$. Scaling is applied to both training and testing apple dataset before applying the SVM package to avoid the attribute of the greater numeric range dominate the smaller one. 

*Step 3*: Perform the *K*-fold cross-validation to find the best parameter *C* and γ for 2-fold, 3-fold, 4-fold, 5-fold and 10-fold.

*Step 4*: Train the whole training set using the parameter *C* and γ obtained in Step 3.

*Step 5*: Test the apple dataset. 

Where, $\;K\left(x,y\right)=e^{-\gamma {\left|\left|x-y\right|\right|}^{2}}$ are the RBF kernel function, γ is the kernel parameter and *C* is the penalty parameter.

#### 3.2.2. Softmax Classifier

The Softmax classifier is one of the most commonly-used logistic regressions for multi-class classification. The Softmax classifier has been verified as a fast training classifier [44,45]. The probabilities for each class label are calculated using the cross-entropy loss function expressed in (2):

(2) $$L_{i}=-f_{y_{i}}+log\underset{j}{\sum }e^{f_{j}}$$

where, $\;f_{j}$ is the *j*-th element for the vector of class scores f.

#### 3.2.3. K-Nearest Neighbors Classifier

Another most widely used classifier in image recognition method is the K-Nearest Neighbors (KNN) [40]. KNN classifier is an instance-based learning classifier where the hypotheses are constructed directly from the training instances. The KNN classifier is simple and easy to implement for classification. KNN classifies an object by referring to the feature similarity where the object is assign to the class based on the majority vote among its *k* nearest neighbors. Where the *k* value is typically a small positive integer. 

### 3.3. Experimental Setup

All the experiments were implemented using MATLAB R2017b on Windows 10 Pro and an Intel Core i7 processor (3.40 GHz) with 8.00 GB RAM. An apple image dataset is created to evaluate the performance of the three methods. The dataset was collected using vision sensors and via the Google search engine with several keywords such as “fresh+apple”, “healthy+apple”, “apple+desease”, “damage+apple”, “defect+apple” and “low+grade+apple+fruit”. Some of the apple images were collected from the Google search engine due to difficulties in obtaining images of various types of apple defect. Most of the images in the dataset are clean without or very few cluttered environments. The position of the apple in the image is at the center and occupied most of the image. This is similar to the apple images captured on the conveyer belt during the classification process. 

**Figure 4:**
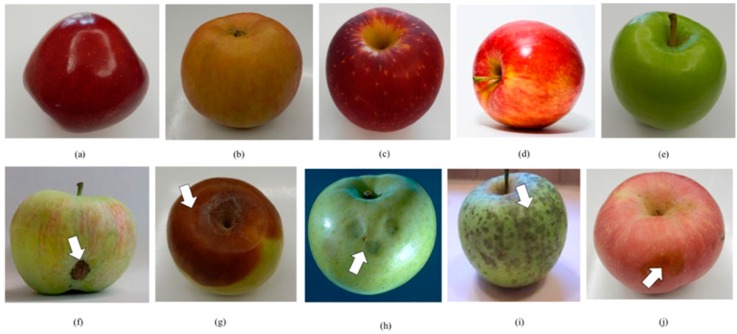
Examples of apple images in the dataset. The first row is non-defective apples and the second row is defective apples: (**a**) Red Delicious, (**b**) Gala, (**c**) Fuji, (**d**) Honeycrisp, (**e**) Granny Smith, (**f**) Scab, (**g**) Rot, (**h**) Cork Spot, (**i**) Blotch, (**j**) Bruise.

Generally, the dataset consists of 550 apple images classified into two categories of non-defective and defective. The apple images with stem end (370 images) and calyx (61 images) which are the natural parts of apple are also included in the dataset for both categories. The resolution for all images is 900 × 700. In this dataset, 76 images are apples with the yellow-white flecks skin type. The properties of the dataset are shown in Table 1. The apple images in the dataset are composed of 275 non-defective apples and 275 defective apples as shown in Table 1. The images of the non-defective apples were collected from five different apple cultivars which are *Red Delicious*, *Gala*, *Fuji*, *Honeycrisp* and *Granny Smith*. On the other hand, the defective category comprises five groups of defects namely *Scab*, *Rot*, *Cork Spot*, *Blotch* and *Bruise*. The defective category includes variations of obvious and unobvious defects with different types, severity, region and size. A sample of the dataset is shown in Figure 4. The complexity of the dataset can be seen in the figure since it consists of various cultivars, types and color of apples with non-defective as well as numerous kind and severity of defect with different poses and angles. The dataset is available online at https://github.com/AsIsmail/Apple-Dataset. 

In this paper, the *K*-fold cross-validation is used to evaluate the proposed vision-based apple classification. There are five *K*-fold cross-validations tested in the experiment namely; 2-fold, 3-fold, 4-fold, 5-fold and 10-fold. The *K*-fold cross-validation will randomly partition the dataset into *K* number of folders. Every fold will have virtually the same number of class distribution. One of the folders will be used for validation while the remaining K−1 folder will be used for training. This process is repeated *K* times until each of the folder is used exactly once as validation set. Finally, the average results from the *K* experiments is calculated.

The above experiments are repeated for BOW, SPM and CNN methods in which, popular methods for image classification and object recognition [16]. Their performances are evaluated and compared.

## 4. Results

In this experiment, the *K*-fold cross-validation is applied to evaluate the effect on the number of training samples towards the classification accuracy and computational time. The classification performance of all methods are measured in terms of sensitivity, specificity and accuracy which is defined as follows:

(3) Sensitivity (SE)=TPR/(TPR+FNR),

(4) Specificity (SP)=TNR/(FPR+TNR),

(5) Accuracy=(TPR+TNR)/(TPR+FPR+TNR+FNR),

where true positive rate (TPR) represents defective apples correctly classified as defective apples, true negative rate (TNR) is the non-defective apples correctly classified as non-defective apples, false positive rate (FPR) is the defective apples incorrectly classified as non-defective apples and false negative rate (FNR) are the non-defective apples incorrectly classified as defective apples. The average results for *K*-fold cross-validation are presented in Table 2.

Following the *K*-fold cross-validation for the 2-fold experiment, a total number of 275 images from the defective class and 275 images from the non-defective class are divided into two equal parts for training and testing. Each part consists of 137 images and 138 images for the defective and non-defective classes, respectively. In Table 2, the classification performances are led by the SPM method for all tested folds. For 2-fold experiment, the SPM method achieved 93.27% classification accuracy. The SPM method correctly classified 131 out of 138 images of non-defective apples followed by CNN (119 images) and BOW (109 images). For the defective images, the SPM method correctly classified 126 out of 138 images of defective apples followed by CNN (118 images) and BOW (109 images). The SPM method took approximately 0.21 s to classify the defective and non-defective apples while CNN took 0.13 s and BOW took 17.48 s. The BOW method requires the longest time to classify between the defective and non-defective apples, nearly 83 times longer compared to the SPM method, whereas the SPM method only took less than 0.08 s longer than CNN.

The accuracy of all methods are increased with the increment number of folds as presented in Table 2. To obtained the most suitable classifier for all the methods, 10-fold cross-validation is applied and the obtained performances are compared. Three classifier were utilized; SVM, Softmax and KNN. The results for 10-fold cross-validation are averaged for each classifier and presented in Table 3.

The 10-fold cross-validation experiment divided the 275 images from the defective class and 275 images from the non-defective class into 10 equal parts. Nine parts are used for training and one for testing. Each part consists of 28 or 27 images of the defective and non-defective classes. From Table 3, the SVM classifier gives the best performance for all the methods: BOW, CNN and SPM. The SPM with SVM classifier achieved a 98.15% classification accuracy by correctly classifying all the non-defective apple images and correctly classifying 26 out of 27 defective images. This is followed by CNN with the SVM classifier which achieved a 94.44% classification accuracy. The CNN with SVM classifier correctly classified 26 of the non-defective apples and correctly classified 25 out of 27 of the defective apple images. A similar performance is achieved by SPM with Softmax classifier, in which CNN with SVM classifier slightly outperforms the SPM with Softmax by a 1.85% classification accuracy. The SPM with Softmax classifier obtained 92.59% accuracy which correctly classified all the non-defective images and correctly classified 23 out of 27 defective images. Nevertheless, a significance difference is observed between the SVM and Softmax classifier in the BOW method, in which the SVM classifier obtained 87.04% while the Softmax classifier only achieved 18.52% classification accuracy. The BOW with SVM classifier correctly classified 23 of the non-defective images and 24 out of 27 defective images, while BOW with the Softmax classifier correctly classified only eight non-defective images and two out of 27 defective images tested. The KNN classifier on the other hand, has the lowest performance for all the methods which is below 60.00% classification accuracy. In terms of computational time, the Softmax classifier is the fastest classifier during the training. This is followed by the SVM classifier with a minimal time difference of less than 0.76 s with SPM, 0.28 s with CNN and 21.40 s for the BOW method. During the testing, the SVM classifier is the fastest, followed by the Softmax classifier at classifying between the defective and non-defective apples. However, the KNN classifier requires the longest time for both training and testing of all the methods. Among the classifiers tested for the BOW, SPM and CNN method, the SVM classifier yields the highest classification accuracy.

## 5. Discussion

**Figure 5:**
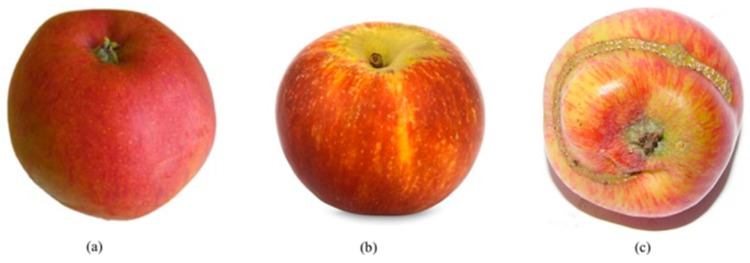
(**a**) Calyx (**b**) stem end (**c**) defect.

**Figure 6:**
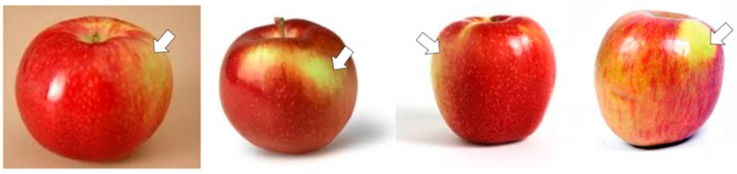
Examples some of the detection failures on bright-skinned apples with yellow-white flecks images indicated by the arrows.

Overall, the SPM with SVM classifier outperformed others in all five *K*-fold cross-validation experiments; 2-fold, 3-fold, 4-fold, 5-fold and 10-fold. The SPM with SVM classifier obtained an average classification accuracy of 6.04% over CNN and 11.58% over the BOW method. Furthermore, the SPM with SVM classifier is able to achieve more than 90% accuracy for both sensitivity and specificity for all tested folds. This indicates that the spatial information in the SPM is very important to discriminate between the defects and stem ends or calyxes. The stem ends and calyxes are the natural parts of the apple located at the top and bottom of the apple while defects can be located at various positions and sizes. However, stem ends or calyxes may be misclassified as defects as they can appear similar to blemish defects, as shown in Figure 5. The spatial information helps in distinguishing these elements although they may be composed by the same visual patterns but with different spatial compositions. Hence, the spatial information is crucial to classify whether the patch is a stem end, calyx, a patch-like blemish or another defect. The false negative classification in which a non-defective apple is misclassified as defective has often been observed on apples with bright skin and apples with yellow-white flecks. Examples of these apples are shown Figure 6. 

**Figure 7:**
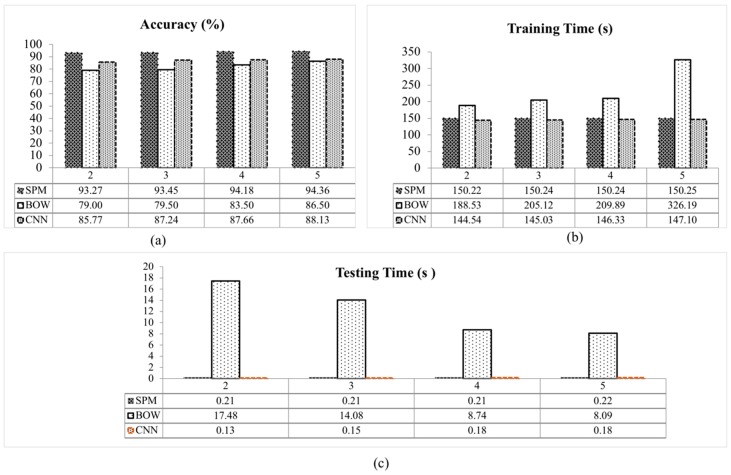
Evaluation on the effect of number of folds on: (**a**) classification accuracy, (**b**) training time and (**c**) testing time for each method with SVM classifier.

A graphical comparison between the classification accuracy of BOW, SPM and CNN with SVM classifier for each fold is presented in Figure 7a. The accuracy of all methods increased with the increment of folds. This is because the number of training images is increased with the increasing number of folds. The SPM with SVM classifier achieved a classification accuracy of 93.27% for 2-fold, 93.45% for 3-fold, 94.18% for 4-fold and 94.36% of 5-fold. A lower classification accuracy for all folds is observed in the CNN and BOW methods. The CNN with SVM classifier presents a slightly lower performance compared to SPM with SVM classifier due to the CNN requirement of having fixed-size input images. In this work, the input images to the CNN method were scaled from the original of 900 × 700 pixels to 227 × 227 pixels. Also, the CNN method requires a large number of images for training to obtain a high classification accuracy [30]. However, in this experiment a limited number of images (550 images) were utilized. These reasons reduce the recognition accuracy of the CNN method [27]. As presented in Figure 7a, the classification accuracy of the SPM with SVM classifier exceeds the others for all tested folds.

In the same experiment, the time taken during training and testing for all folds are also measured as presented in Table 2. From these results, the training time for all methods became longer as the number of folds increased. This is because when the number of folds is increased, the number of training images is also increased. 

Among the methods, BOW takes the longest testing time, between 8.09 s to 17.48 s, whereas the CNN method is the fastest, followed by the SPM method. The testing time for these methods are separated by less than a second for all tested folds. The training and testing time for all methods are shown in Figure 7b,c. Overall, BOW took the longest time to train and classify the images compared to the SPM and CNN methods. The is due to the high computational cost in vector quantization step in the BOW method as it requires a large number of feature vector to recognize an object. The training and testing time for SPM and CNN methods are nearly similar for all tested folds.

**Figure 8:**
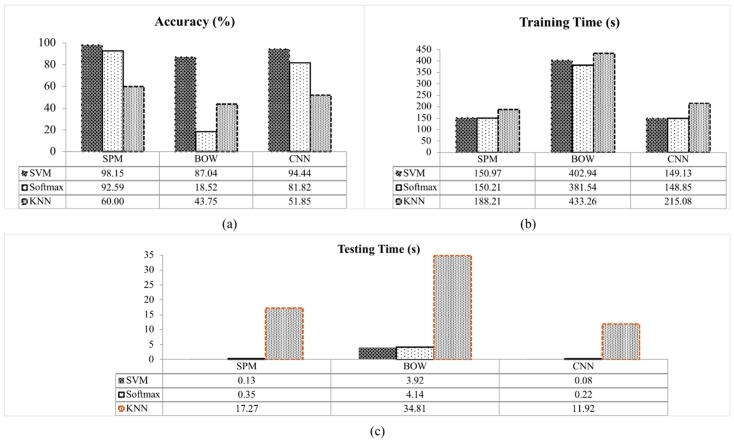
Performance of each method for different classifier (**a**) classification accuracy, (**b**) training time and (**c**) testing time.

In this work, the performance between classifiers for each method are also evaluated. The overall performance for each method with different classifiers is illustrated in Figure 8. Figure 8a shows that the SVM classifier achieved the highest accuracy compared to the others. This is because the SVM classifier is suitable to be applied on a small sample data with high feature vector dimensionality [46]. Since the SVM classifier has less problems with dimensionality, the high dimensionality of features vectors doesn’t degrade it’s effectiveness [20]. This contributes to the success of detection on all methods. The SPM with SVM classifier achieved the classification accuracy of 98.15%, BOW 87.04% and CNN 94.44% for 10-fold experiment. A lower classification accuracy can be seen in Softmax and KNN classifier. The classification accuracy of the KNN classifier is the lowest for all the methods with BOW 43.75%, CNN 51.85% and SPM 60.00%. The KNN classifier determine the classes by calculating the distance between the data based on the simple vote majority system [47]. However, the distance of the data from different classes may be similar [40], thus, increases the misclassification. The Softmax classifier classifies the SPM and CNN features with a classification accuracy of 92.59% and 81.82%, respectively. The lowest accuracy for the Softmax method is observed for the BOW method with an accuracy of 18.52%. The unstructured BOW method where the spatial layout information was discarded contributed to this low classification accuracy [18]. In the BOW method, the images are described by counting the frequency of occurrence for each visual pattern (visual words) and used as a feature for training the Softmax classifier. Averaging the visual words in the Softmax classifier [45] results in the detection failures between the defective and non-defective apples in the BOW method. In contrast, the accuracy of the BOW method was significantly improved with the SVM classifier compared to others. The BOW with SVM classifier achieved 87.04% accuracy. This indicates that the SVM classifier is suitable to classify the BOW features. In the SVM classifier, each of the data instances are represented in the form of vectors of real numbers. The SVM classifier also considered the compositionality of each data instance. 

The training and testing time for all classifier tested are shown in Figure 8b,c. Among the classifiers, KNN taken the longest time for training and to classify the dataset. The KNN classifier is computationally intensive as it stores all the training data and compares the extracted features on the test images with each training data for classification [40]. In contrast, the Softmax classifier is low in complexity and a fast classifier during training as the Softmax classifier does not required calculating the probability of each training sample during training [45]. This is followed by the SVM classifier with minimal time difference between the training time of Softmax. 

On the other hand, during testing, the SVM classifier is the fastest classifier because of the data scaling in the SVM package that avoids the attribute with the greater numeric range dominating a smaller one [43]. This reduces the time complexity. Finally, the experimental results show that the SPM with SVM classifier is the most suitable and effective method for vision-based apple classification.

## 6. Conclusions

A vision-based apple classification for smart manufacturing is proposed to optimize production based on current production data about defective and non-defective apples through data analytics and visualization. The proposed system converts the synthesized data into actionable knowledge for automated inspection, sorting and further analytics. Three image recognition methods namely the BOW, SPM and CNN methods were tested with the SVM, Softmax and KNN classifiers in the proposed vision-based apple classification application. These methods analyzed and then classified the apple images captured by the visual sensors as defective or non-defective. Experimental results showed that SPM with SVM classifier achieved the highest performance among all methods. The recognition accuracy of the SPM with SVM classifier is 98.15%, BOW with SVM classifier obtained 87.04% whereas CNN with SVM classifier achieved 94.44% for a 10-fold cross-validation. Overall, the recognition accuracy of the SPM with SVM classifier outperformed BOW and CNN by 7.32% and 3.71%, respectively. The results show that SPM is robust to spatial features translation and can efficiently distinguish and classify defective and non-defective apples. In terms of testing time, CNN is the fastest among all methods, followed by the SPM and BOW. However, the SPM only took 0.05 s longer than CNN in classifying the apple images. Despite the highest recognition accuracy of the SPM, this method misclassified non-defective apples with yellow-white flecks skin as defective apples. Although the SPM method is less effective in detecting non-defective apples with yellow-white flecks skin, the SPM method classified almost all the defective types; including obvious and unobvious defects with different severity, region and size. The results show that the proposed vision-based apple classification systems can be used in assisting the decision-making process in apple product manufacturing industries. Therefore, in the future, this work can be extended to include other feature detection techniques. The SPM method combined with features such as Speeded-Up Robust Features (SURF), Binary Robust Invariant Scalable (BRISK) and Binary Robust Independent Elementary Features (BRIEF) may help improve the current results. This is possible since these features can improve the detection on the apple skin images as they will detect points of interest or changes in any different property of the texture elements that appear on the apple skin.

## Figures and Tables

**Table 1:** Details of the characteristics in apple dataset.

Non-Defective		Defective	
Cultivars	Total	Types	Total
*Red Delicious*	53	*Scab*	71
*Gala*	57	*Rot*	79
*Fuji*	58	*Cork Spot*	61
*Honeycrisp*	56	*Blotch*	32
*Granny Smith*	51	*Bruise*	32
Total	275	Total	275

**Table 2:** Confusion matrix of *K*-fold cross-validation for each method with SVM classifier.

Class	*K*-Fold							
	2		3		4		5	
**BOW**								
	**Defective**	**Non-Defective**	**Defective**	**Non-Defective**	**Defective**	**Non-Defective**	**Defective**	**Non-Defective**
Defective	109	29	78	14	57	12	49	6
Non-defective	29	109	24	68	10	59	9	46
*Sensitivity*	0.79		0.77		0.85		0.85	
*Specificity*	0.79		0.83		0.83		0.88	
*Accuracy* (%)	79.00		79.50		83.50		86.50	
*Training time* (s)	188.53		205.12		209.89		326.19	
*Testing Time* (s)	17.48		14.08		8.74		8.09	
**CNN**								
	**Defective**	**Non-Defective**	**Defective**	**Non-Defective**	**Defective**	**Non-Defective**	**Defective**	**Non-Defective**
Defective	118	20	80	12	58	11	49	6
Non-defective	19	119	11	81	6	63	7	48
*Sensitivity*	0.86		0.88		0.90		0.87	
*Specificity*	0.86		0.87		0.84		0.89	
*Accuracy* (%)	85.77		87.24		87.66		88.13	
*Training time* (s)	144.54		145.03		146.33		147.10	
*Testing Time* (s)	0.13		0.15		0.18		0.18	
**SPM**								
	**Defective**	**Non-Defective**	**Defective**	**Non-Defective**	**Defective**	**Non-Defective**	**Defective**	**Non-Defective**
Defective	126	12	85	7	63	6	51	4
Non-defective	7	131	5	87	3	66	2	53
*Sensitivity*	0.95		0.95		0.96		0.96	
*Specificity*	0.92		0.92		0.92		0.93	
*Accuracy* (%)	93.27		93.45		94.18		94.36	
*Training time* (s)	150.22		150.24		150.24		150.25	
*Testing Time* (s)	0.21		0.21		0.21		0.22	

**Table 3:** Comparison of confusion matrix for BOW, CNN and SPM method with different classifiers.

Class	Classifier					
	SVM		Softmax		KNN	
**BOW**						
	**Defective**	**Non-Defective**	**Defective**	**Non-Defective**	**Defective**	**Non-Defective**
Defective	24	3	2	25	20	7
Non-defective	4	23	19	8	24	3
*Sensitivity*	0.86		0.10		0.46	
*Specificity*	0.88		0.24		0.33	
*Accuracy* (%)	87.04		18.52		43.75	
*Training time* (s)	402.94		381.54		433.26	
*Testing Time* (s)	3.92		4.14		34.81	
**CNN**						
	**Defective**	**Non-Defective**	**Defective**	**Non-Defective**	**Defective**	**Non-Defective**
Defective	25	2	22	5	23	4
Non-defective	1	26	5	22	22	5
*Sensitivity*	0.96		0.82		0.51	
*Specificity*	0.93		0.82		0.56	
*Accuracy* (%)	94.44		81.82		51.85	
*Training time* (s)	149.13		148.85		215.08	
*Testing Time* (s)	0.08		0.22		11.92	
**SPM**						
	**Defective**	**Non-Defective**	**Defective**	**Non-Defective**	**Defective**	**Non-Defective**
Defective	26	1	23	4	24	3
Non-defective	0	27	0	27	19	9
*Sensitivity*	1		1		0.56	
*Specificity*	0.96		0.87		0.32	
*Accuracy* (%)	98.15		92.59		60.00	
*Training time* (s)	150.97		150.21		188.21	
*Testing Time* (s)	0.13		0.35		17.27	
